# The chloroplast genome of *Rosa rugosa × Rosa sertata* (Rosaceae): genome structure and comparative analysis

**DOI:** 10.1590/1678-4685-GMB-2021-0319

**Published:** 2022-10-03

**Authors:** Yuan Niu, Yanyan Luo, Chunlei Wang, Qiong Xu, Weibiao Liao

**Affiliations:** 1 Gansu Agricultural University, College of Horticulture, Lanzhou, China.; 2 Lanzhou Agro-technical research and Popularization Center, Lanzhou, China

**Keywords:** Rosaceae, Rosa rugosa × Rosa sertata, chloroplast genome, phylogenetic relationship

## Abstract

*Rosa rugosa × Rosa sertata*, which belongs to the family Rosaceae, is one of the native oil-bearing roses in China. Most research has focused on its essential oil components and medicinal values. However, there have been few studies about its chloroplast genome. In this study, the whole chloroplast genome of *R. rugosa × R. sertata* was sequenced, analyzed, and compared to other genus *Rosa* species. The chloroplast genome of *R. rugosa × R. sertata* is a circular structure and 157,120 bp in length. The large single copy and small single copy is 86,173 bp and 18,743 bp in size, respectively, and the inverted repeats are 26,102 bp in size. The GC content of the whole genome is 37.96%, while those of regions of LSC, SSC, and IR are 35.20%, 31.18%, and 42.73%, respectively. There are 130 different genes annotated in this chloroplast genome, including 84 protein coding genes, 37 tRNA genes, 8 rRNA genes, and 1 pseudogene. Phylogenetic analysis of 19 species revealed that *R. rugosa × R. sertata* belong to the Sect. *Cinnamomeae*. Overall, this study, providing genomic resources of *R. rugosa × R. sertata*, will be beneficial for species identification and biological research.

*Rosa*, a typical genus of the Rosaceae family, is widely distributed over the northern hemisphere (Rehder, 1949; Christenhusz et al., 2017). Traditionally, it has a wide range of uses, such as food, decoration, medicine, perfume industry, and ecological conservation (Wang et al., 2012; Cheng et al., 2016; Patel, 2017). *Rosa rugosa × Rosa sertata*, belonging to the genus *Rosa* of *Rosaceae*, is an important economic tree species in China. It is commonly called Kushui rose because it is mainly planted in Kushui, Lanzhou city of Gansu Province for production of dried flower bud tea, jam, rose essential oil, hydrosol, and other products. Among them, essential oil extraction products have appeared in the international market since the late 1980s. Studies on the composition of its essential oils show that it has high alcohol content, including citronellol, geraniol and farnesol (Son and Lee, 2012; Wu Y et al., 2020). It is worth noting that the relative content of citronellol is nearly half. Moreover, the decoction made from its flower tea has been proved to have anti-cancer antioxidant activity (Liu et al., 2018). A new kind of polysaccharide, which can be used as a safe immune regulator in the field of medicine or functional food, was found from the waste of the processing of its essential oil (Wu M *et al.*, 2019). The evolutionary origin of most roses remains elusive, and the species in this study is not the exception, although it is recorded as a natural hybrid with the corresponding Latin name. Most puzzling is that there is no direct genetic evidence of its hybridization background. 

The chloroplast, as one of the plants' organelles, plays an important role in maintaining life on earth by the process of conversion of solar energy into carbohydrates through photosynthesis and the release of oxygen (Daniell et al., 2016). Therefore, various essential genes for carbon fixation and metabolite synthesis exist in chloroplast genome. The common chloroplast genomes, ranging from 120 to 170 kb in size, generally encode 120 to 130 genes. They are usually composed of four parts, namely a large single copy (LSC) region, a small single copy (SSC) region and a pair of reverse repeat regions separating the first two parts (Bendich, 2004). For phylogeny and population genetics, it is necessary to study chloroplast genomes, because of their conservative gene structure and base content, and their ability to solve the relationship at a lower classification level (Wicke et al., 2011). In recent years, the progress of next generation sequencing technology provides researchers with faster and cheaper methods to obtain chloroplast genome information. In this study, the complete chloroplast genome of *R. rugosa × R. sertata* was first obtained by high-throughput sequencing technology and compared with other species within the genus of *Rosa*. 

Healthy and mature leaves of *R. rugosa × R. sertata* were collected from Gansu Agricultural University (36°09′N, 103°70′E, Lanzhou, Gansu, China) and were preserved in liquid nitrogen and then stored in an Ultra-low temperature freezer until DNA extraction. Total genomic DNA was extracted from sampled leaves using a Plant Genomic DNA kit (TIANGEN, Beijing, China) following the manufacturer’s instructions. The isolated genomic was used to prepare high-throughput DNA sequencing libraries with Illumina V3 kit (catalog number:ND607 Vazyme), and library products corresponding to 300-350bps were enriched, quantified and sequenced on Novaseq 6000 sequencer (Illumina) with PE 150 model. Generated 17,902,347 paired-end raw reads and the sequencing data was first filtered by Trimmomatic (version 0.36), low-quality reads were discarded and the reads contaminated with adaptor sequences were trimmed. The clean reads and reference sequence as *R. acicularis* (Chen et al., 2019) (GenBank accession no. MK714016.1) were used to extract chloroplast-like reads, which aligned to the database built by Genepioneer Biotechnologies (Nanjing, China) using Bowtie2 v2.2.4 (Langmead and Salzberg, 2012) and SPAdes v3.10.1 (Bankevich et al., 2012). Then, the sequences with the cp-like reads were assembled with NOVOPlasty (Dierckxsens et al., 2017). Annotation of the assembled chloroplast sequence was conducted with two methods. Firstly, the CDS, rRNA and tRNA were predicted with Prodigal v2.6.3 (Hyatt et al., 2010), hmmer v3.1b2 (Prakash et al., 2017) and Aragorn v1.2.38 (Laslett and Canback, 2004), respectively. Secondly, blast v2.6 (Johnson et al., 2008) was used to compare the gene sequences of the assembled one and the reference species. To determine the final annotation, the above two results were manually checked to remove the redundant and determine the multiple exon boundaries. A circular map of *R. rugosa × R. sertata* plastid genome was generated using the Chloroplot program (Zheng et al., 2020). 

The whole chloroplast genome sequence of *R. rugosa × R. sertata* was determined and deposited to GenBank under accession number: MT845214. The size of the complete chloroplast genome is 157,120 bp, near other *Rosa* chloroplast genome level. It displayed a typical quadripartite structure, possessing a LSC region (86,176 bp), an SSC region (18,743 bp) and a pair of IR region (52,204 bp) (Figure 1). The overall GC content is 37.22%, and the order of GC content in different regions is 42.73% in IR regions, 35.20% in LSC region and 31.18% in SSC region (Table S1). It is a normal phenomenon that the highest GC content exists in the IR regions in different plants. There has been studies that show that such GC skewness can be indicators of replication origins, replication terminals, DNA lead chains or lag chains (Tillier and Collins, 2000; Necşulea and Lobry, 2007). 

**Figure 1 -:**
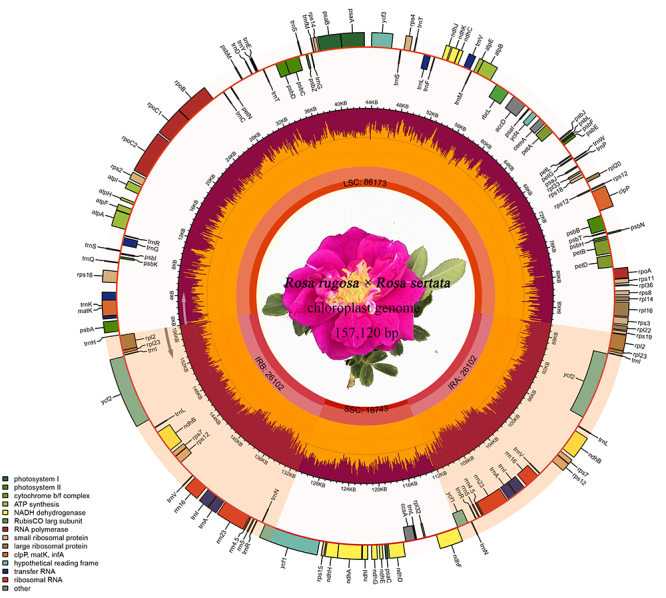
Gene map of the *R. rugosa* × *R. sertata* chloroplast genome. The genes with different functional classification are color coded and the pseudogene is marked asterisks. The genes are shown inside and outside the outermost layer represented with transcription directions clockwise and counterclockwise, respectively. The GC content is depicted as the proportion of the shaded parts of each section in the innermost layer.

A total of 130 functional genes were detected in *R. rugosa × R. sertata* chloroplast genome, including 84 protein-coding genes, 37 tRNA genes, and 8 rRNA genes. In the IR regions, there were 12 protein-coding genes, 14 tRNA genes and 8 rRNA genes. In the LSC and SSC region, there were 60 and 12 protein-coding genes, and 22 and 1 tRNA genes, respectively (Table 1, Table S2). In addition, the *ycf1* was interpreted as pseudogene in our study as it contains several internal stop codons. Studies have shown that splicing introns are the hallmark of eukaryotic genes (Niu and Yang, 2011). In this study, 22 genes with intron structure were detected in this chloroplast genome, including 8 tRNA genes and 13 protein-coding genes (Table S3). Among them, 11 genes are in the LSC regions: 1 gene in the SSC region and 10 genes in the IR region. Genes *ycf3* and *clpP* contain two introns, which is consistent with other chloroplast genomes (Li et al., 2018; Xue et al., 2019; Liu et al., 2020). 

**Table 1 -:** List of genes annotated in the R. rugosa × R. sertata chloroplast genome.

Category	Gene group	Gene name	Number
Photosynthesis	photosystem I	psaA,psaB,psaC,psaI,psaJ	5
	photosystem II	psbA,psbB,psbC,psbD,psbE,psbF,psbH,psbI,psbJ,psbK,psbL, psbM,psbN,psbT,psbZ	15
	NADH dehydrogenase	ndhA*,ndhB*(2),ndhC,ndhD,ndhE,ndhF,ndhG,ndhH,ndhI, ndhJ,ndhK	12
	cytochrome b/f complex	petA,petB*,petD*,petG,petL,petN	6
	ATP synthase	atpA,atpB,atpE,atpF,atpH,atpI	6
	Large subunit of rubisco	rbcL	1
	photochlorophyllide reductase	-	
Self-replication	large ribosomal subunit	rpl14,rpl16*,rpl2*(2),rpl20,rpl22,rpl23(2),rpl32,rpl33,rpl36	11
	small ribosomal subunit	rps11,rps12**(2),rps14,rps15,rps16*,rps18,rps19,rps2,rps3, rps4,rps7(2),rps8	14
	RNA polymerase	rpoA,rpoB,rpoC1*,rpoC2	4
	Ribosomal RNAs	rrn16(2),rrn23(2),rrn4.5(2),rrn5(2)	8
	Transfer RNAs	trnA-UGC*(2),trnC-GCA,trnD-GUC,trnE-UUC,trnF-GAA, trnG-GCC*,trnG-UCC,trnH-GUG,trnI-CAU(2),trnI-GAU*(2), trnK-UUU*,trnL-CAA(2),trnL-UAA*,trnL-UAG,trnM-CAU, trnN-GUU(2),trnP-UGG,trnQ-UUG,trnR-ACG(2),trnR-UCU, trnS-GCU,trnS-GGA,trnS-UGA,trnT-GGU,trnT-UGU, trnV-GAC(2),trnV-UAC*,trnW-CCA,trnY-GUA,trnfM-CAU	37
Other genes	Maturase	matK	1
	Protease	clpP**	1
	Envelope membrane protein	cemA	1
	Acetyl-CoA carboxylase	accD	1
	c-type cytochrome synthesis gene	ccsA	1
	Translation initiation factor	-	0
	other	-	0
Genes of unknown function	Conserved hypothetical chloroplast ORF	#ycf1,ycf1,ycf2(2),ycf3**,ycf4	6
Total			130

Notes: Gene*: Gene with one introns; Gene**: Gene with two introns; #Gene: Pseudo gene; Gene(2):Number of copies of multi-copy genes; - : Nonexistent gene

A total of 37 long repeats were detected by REPuter software, including 16 forward repeats, 19 palindromic repeats, and 2 reverse repeats (Figure S1). More than half of the long repeats (51%) were distributed in intergenic spaces (IGSs), 10.81% in both genes and IGSs, and 37.83% in genes. In addition, the distribution of these repeats in different regions is distinct. The number of repeats in regions of LSC, SSC, IRa, and IRb is 19, 3, 13, and 13, respectively. Some repeats, such as genes of *ycf1*, *ycf2*, *ndhB*, *trnG-GCC*, and *trnS-UGA*, existed in two regions simultaneously. In total, 260 SSRs were detected in *R. rugosa × R. sertata* chloroplast genome by software MISA. Among them, 65.55%, 19.33% and 15.13% were in the regions of LSC, IR, and SSC, respectively. Besides, from the perspective of the relationship with location of genes, 52.52%, 34.45%, and 13.03% were found in the IGSs, coding regions and introns, respectively. Types with a number of 20 or more were A (8), T(8), and T(9), while types with numbers between 5 and 20 were A(9), A(10), T(10), T(11), TA(5), TAA(3), TTA(3), and TTC(3). The number of other types was less than 5. The frequencies of mononucleotide, dinucleotide, trinucleotide, tetranucleotide, pentanucleotide, and hexanucleotide were 62.69%, 5.00%, 26.92%, 4.23%, 0.38%, and 0.77%, respectively. Among the identified mononucleotide SSRs, A/T types (92.64%) was dominant compared with G/C types (7.36%). 

Gene flow between species or genetic diversity within a species is often measured by comparison of the chloroplast sequences. To determine differences in the chloroplast genome sequences of *R. rugosa*, *R. odorata var. gigantea*, *R. multiflora*, *R. luciae*, *R. canina* and *R. rugosa × R. sertata*, sequence identity was calculated for these species’ chloroplast sequence using the online program mVISTA with *R. chinensis cultivar Old Blush* as a reference (Figure S2, Table S4). Consistent with other studies, the region of greatest divergence is LSC, in which the noncoding regions possess higher divergence than coding regions. The chloroplast genome of *R. rugosa × R. sertata* is closer to *R. rugosa*, and the significant variation between them could be found in the intergenic regions of *psbM-trnD*, *trnD-trnY*, *rbcL-accD*, *petB-petD*, *petD-rpoA*, *rps3-rpl22*, *trnL-ndhB* and *ndhF-rpl32* (Supplementary Data). It would be considered valuable to utilize the identification of these higher-resolution loci for species identification.

In the long term of evolution, the change of the IR region at the borders plays a critical role. In our study, the genetic architecture of seven *Rosa* genomes was mapped at the junction of the IR region, LSC region, and SSC region by IRscope (Figure S3). Gene location and gene order were relatively conservative in *Rosa*. In *R. canina*, *R. odorata*, *R. rugosa*, *R. chinensi*s, and *R. rugosa × R. sertata*, the codding region of *ycf1* was at the boarder of SSC/IRa, and spanned the SSC and IRa region, while in *R. lucieae* and *R. multiflora*, it was at the boarder of SSC/IRb and spanned the SSC and IRb region. It is noteworthy that in *R. rugosa* and *R. rugosa x R. sertata*, the pseudogene *ycf1* was located in IRb, while in *R. lucieae* and *R. multiflora*, it was located in IRa. The mutation region of pseudogene ycf1 in IRa/SSC or IRb/SSC region was 1106-1111bp. 

The phylogenetic analysis was performed based on complete chloroplast genome sequences from 19 taxa, including 18 *Rosa* species and one outgroup (*Vitis vinifera,* MN561034.1), all of which were downloaded from the NCBI database except the *R. rugosa × R. sertata*. All the sequences from these 19 species were aligned by MAFFT v 7.455 (Katoh and Standley, 2013) and trimmed by trimAl (Capella-Gutiérrez et al., 2009). A maximum likelihood (ML) analysis was performed by IQtree (Nguyen et al., 2015), and a bootstrap test was set with 1000 repetitions. The result of phylogenetic analysis was visualized by MEGA v7.0 (Kumar et al., 2016) (Figure 2). The chloroplast genomes play a significant role in understanding the evolutionary relationship and history of plant species (Jansen et al., 2007). Here, as expected, 14 species from the *Rosa* genus formed a monophyletic clade composed of seven branches, which were consistent with the seven subgroups obtained by morphological classification. *R. rugosa × R. sertata* was mostly related to *R. rugosa*, with bootstrap support value of 100%. They all belong to the Sect. *Cinnamomeae*. The availability of a completed *R. rugosa × R. sertata* chloroplast genome sequence will provide useful information for the phylogenetic study among *Rosa*. 

**Figure 2 -:**
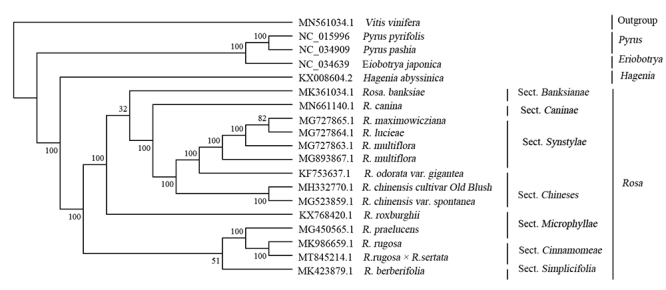
Maximum likelihood (ML) phylogenetic tree of 18 species of Rosaceae constructed using their chloroplast genomes. *Vits vinifera* was used as the outgroup.

Overall, the complete chloroplast genome of *R. rugosa × R. sertata*, an endemic oil-bearing rose species in China, was firstly reported and analyzed. The characteristics of quadripartite structure, genome size, GC content, and gene order of the plastid genome of *R. rugosa × R. sertata* were shown to be similar with that of other genus *Rosa* species. There were 37 long repeats sequences and 260 SSRs detected in this plastid genome. Besides, reconstructed phylogenetic relationships among 19 species found *R. rugosa × R. sertata* to be closely related to *R. rugosa.* These results combined with the comparison with the whole chloroplast genome of other genus Rosa species have provided the worthy information and will bring insight into developing DNA markers suitable for identification of species within this genus.

## Supplementary Material

The following online material is available for this article:

Table S1 - Base composition in the *R. rugosa × R. sertata* chloroplast genome.

Table S2 - The number of genes in the *R. rugosa × R. sertata* chloroplast genome.

Table S3 - The length of exons and introns in genes in the *R. rugosa × R. sertata* chloroplast genome.

Table S4 - Statistics of the chloroplast genomes of *R. rugosa × R. sertata* and five other Rosaceae species.

Figure S1 - Analysis of long repeat sequences and simple sequence repeats (SSRs) in *R. rugosa × R. sertata* chloroplast genome.

Figure S2 - Sequence identity plot of 6 *Rosa* chloroplast genomes by mVISTA.

Figure S3 - Comparison of LSC, SSC and IR regions in chloroplast genomes.

Supplementary Data - Sequence information of eight Intergenic Regions in the *R. rugosa × R. sertata* and *R. rugosa* chloroplast genome.
