# Supplementary material for: The chloroplast genome of Rosa rugosa × Rosa sertata (Rosaceae): genome structure and comparative analysis
Source: Genet Mol Biol. 2022 Oct 3;45(3):e20210319. doi: 10.1590/1678-4685-GMB-2021-0319 (PMC9540792; doi:10.1590/1678-4685-GMB-2021-0319)
Supplement: Table S1 - [file 1415-4757-GMB-45-3-e20210319-s1.pdf]

Genetics and Molecular Biology
https://doi.org/10.1590/1678-4685-GMB-2021-0319

Supplementary material to "The Chloroplast Genome of *Rosa rugosa* × *Rosa sertata* (Rosaceae): Genome Structure and Comparative Analysis"

Table S1 - Base composition in the *R. rugosa* × *R. sertata* chloroplast genome.

Region	A (%)	T (%)	C (%)	G (%)	A+T (%)	G+C (%)
**LSC**	31.70	33.09	18.07	17.13	64.79	35.20
**SSC**	34.48	34.33	16.23	14.95	68.81	31.18
**IRa**	28.56	28.71	22.21	20.52	57.27	42.73
**IRb**	28.71	28.56	20.52	22.21	57.27	42.73
**Total**	31.02	31.76	18.95	18.28	62.04	37.96
