# Supplementary material for: The chloroplast genome of Rosa rugosa × Rosa sertata (Rosaceae): genome structure and comparative analysis
Source: Genet Mol Biol. 2022 Oct 3;45(3):e20210319. doi: 10.1590/1678-4685-GMB-2021-0319 (PMC9540792; doi:10.1590/1678-4685-GMB-2021-0319)
Supplement: Table S2 - [file 1415-4757-GMB-45-3-e20210319-s2.pdf]

Genetics and Molecular Biology
https://doi.org/10.1590/1678-4685-GMB-2021-0319

Supplementary material to "The Chloroplast Genome of *Rosa rugosa* × *Rosa sertata* (Rosaceae): Genome Structure and Comparative Analysis"

Table S2 - The number of genes in the *R. rugosa* × *R. sertata* chloroplast genome.

Region	Numeber of CDS	Numeber of tRNA	Numeber of rRNA	Total
**LSC**	60	22	0	82
**IRb**	6	7	4	17
**SSC**	12	1	0	13
**IRa**	6	7	4	17
