# Supplementary material for: The chloroplast genome of Rosa rugosa × Rosa sertata (Rosaceae): genome structure and comparative analysis
Source: Genet Mol Biol. 2022 Oct 3;45(3):e20210319. doi: 10.1590/1678-4685-GMB-2021-0319 (PMC9540792; doi:10.1590/1678-4685-GMB-2021-0319)
Supplement: Table S3 - [file 1415-4757-GMB-45-3-e20210319-s3.pdf]

Genetics and Molecular Biology
https://doi.org/10.1590/1678-4685-GMB-2021-0319

Supplementary material to "The Chloroplast Genome of *Rosa rugosa* × *Rosa sertata* (Rosaceae): Genome Structure and Comparative Analysis"

Table S3 - The length of exons and introns in genes in the *R. rugosa* × *R. sertata* chloroplast genome.

Gene	Location	Exon I(bp)	Intron II (bp)	Exon II (bp)	Intron II (bp)	Exon III (bp)
**trnK-UUU**	LSC	37	2495	35		
**rps16**	LSC	32	880	229		
**trnG-GCC**	LSC	23	693	48		
**rpoC1**	LSC	435	761	1611		
**ycf3**	LSC	126	779	228	738	153
**trnL-UAA**	LSC	37	546	50		
**trnV-UAC**	LSC	39	596	37		
**rps12**	IRa	114	-	232	538	26
**clpP**	LSC	69	649	291	833	228
**petB**	LSC	6	789	642		
**petD**	LSC	9	719	474		
**rpl16**	LSC	9	968	399		
**rpl2**	IRb	391	681	434		
**ndhB**	IRb	777	676	756		
**rps12**	IRb	232	-	26	538	114
**trnI-GAU**	IRb	42	949	35		
**trnA-UGC**	IRb	38	814	35		
**ndhA**	SSC	552	1217	540		
**trnA-UGC**	IRa	38	814	35		
**trnI-GAU**	IRa	42	949	35		
**ndhB**	IRa	777	676	756		
**rpl2**	IRa	391	681	434		
