# Supplementary material for: The chloroplast genome of Rosa rugosa × Rosa sertata (Rosaceae): genome structure and comparative analysis
Source: Genet Mol Biol. 2022 Oct 3;45(3):e20210319. doi: 10.1590/1678-4685-GMB-2021-0319 (PMC9540792; doi:10.1590/1678-4685-GMB-2021-0319)
Supplement: Table S4 - [file 1415-4757-GMB-45-3-e20210319-s4.pdf]

Genetics and Molecular Biology
https://doi.org/10.1590/1678-4685-GMB-2021-0319

Supplementary material to “The Chloroplast Genome of *Rosa rugosa* × *Rosa sertata* (Rosaceae): Genome Structure and Comparative Analysis”

Table S4 - Statistics of the chloroplast genomes of *R. rugosa* × *R. sertata* and five other Rosaceae species.

Species	Total Length (bp)	LSC (bp)	IR (bp)	SSC (bp)	Total	Protein coding genes	tRNA	rRNA	GC%	Accession number in Genbank
R. rugosa* × *R. sertata	157,120	86,173	26,102	18,743	130	84	37	8	38.0	MT845214
R. rugosa	156,999	86,039	26,051	18,858	134	84	41	8	37.0	MK986659
R. odorata var. gigantea	156,634	85,767	26,053	18,761	115	81	30	4	37.2	KF753637
R. multiflora	156,519	85,643	26,058	18,760	114	80	30	4	37.2	MG727863
R. luciae	156,506	85,631	26,058	18,759	114	80	30	4	37.2	MG727864
R. canina	156,501	85,653	26,053	18,742	113	79	30	4	37.3	MN661140
