# Supplementary material for: The chloroplast genome of Rosa rugosa × Rosa sertata (Rosaceae): genome structure and comparative analysis
Source: Genet Mol Biol. 2022 Oct 3;45(3):e20210319. doi: 10.1590/1678-4685-GMB-2021-0319 (PMC9540792; doi:10.1590/1678-4685-GMB-2021-0319)
Supplement: Figure S1 - [file 1415-4757-GMB-45-3-e20210319-s5.pdf]

Genetics and Molecular Biology
https://doi.org/10.1590/1678-4685-GMB-2021-0319

Supplementary material to "The Chloroplast Genome of *Rosa rugosa* × *Rosa sertata* (Rosaceae): Genome Structure and Comparative Analysis"

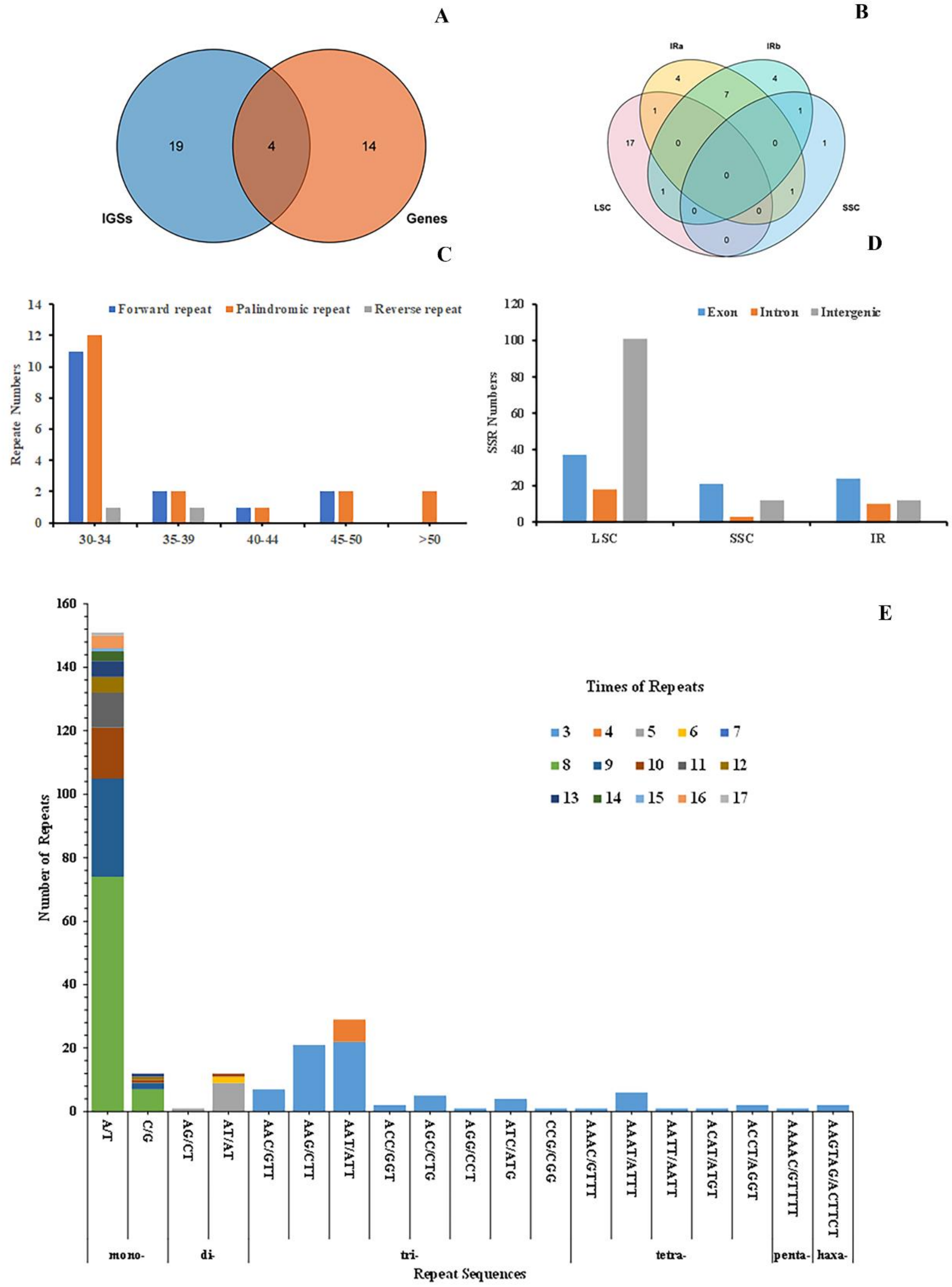

https://doi.org/10.1590/1678-4685-GMB-2021-0319

Figure S1 - Analysis of long repeat sequences and simple sequence repeats (SSRs) in *R. rugosa* × *R. sertata* chloroplast genome. (A) The number of long repeat sequences in genes and IGSs. (B) The number of long repeat sequences in regions of LSC, SSC, IRa and IRb. (C) The number of different types of long repeat sequences detected in the chloroplast genome. Blue, orange, and gray represent forward, palindromic, and reverse repeat, respectively. The horizontal axis represents the types of repeats sequences of different lengths. The vertical axis represents the number of repeats. (D) Number of SSRs in the regions of LSC, SSC, and IR. Blue, orange, and gray represent the sequences of exon, intron, and intergenic, respectively. The horizontal axis represents different regions of the chloroplast genome. The vertical axis represents the number of SSRs. (E) The number of all different types of SSRs detected in the chloroplast genome. Different colors represent different repetitions of the sequence. The horizontal axis represents different types of SSRs. The vertical axis represents the numbers of repeats."
